# Bibliographical Notices

**Published:** 1856-12

**Authors:** 


### BIBLIOGRAPHICAL NOTICES.

*A Treatise on Therapeutics and Pharmacology, or Materia Medica.* By GEORGE B. WOOD, M. D., Late President of the American Medical Association; President of the College of Physicians of Philadelphia; &c. &c. In two volumes. Philadelphia: J. B. Lippincott & Co., 1856.

Perhaps no one, now living, has done more for Medicine in America, than the author of this treatise; and yet the value of his service, apart from the labors of the College and of the Hospital, has consisted rather in the character of his published works, than in their number. Nor do we find in them much evidence of that easy triumph of mind, which some men obtain, by intuitive perception or original invention. All would seem to have been accomplished by untiring research, profound thought, careful observation, and wide experience. Yet the *Dispensatory*, the *Practice*, the *Therapeutics*, might, either of them, furnish solid and substantial matter enough for a "*monumentum œre perennius.*" The first has become an indispensable Lexicon to every medical office and pharmaceutical establishment. The second is pronounced by high foreign as well as American authority, to be the most complete and useful work on the practice of medicine in the English language. The last, which we propose now briefly to notice, is fully worthy of companionship with the others, and will serve as a sort of connecting link between them.

To those familiar with Prof. Wood's former course of Lectures upon Materia Medica, it will be evident that this work is, as announced in the preface, an extension and elaboration of those lectures. The order of subjects is essentially that enunciated in the "Syllabus;" and it is one which could not be easily altered with advantage. In glancing through the work,—not undertaking, of course, to sift its whole contents,—we shall select only a few of those points which afford to us the most peculiar interest; while it may not seem, we hope, the mere wantonness of criticism, to note an occasional subject upon which even these "*verba magistri*" present, to us, some difficulties.

Considering that, with some possible exceptions, the mode of operation of medicines is by absorption into the blood, and not by nervous influence, Prof. Wood lays stress upon "the control of the vital forces over the tissues;" which, he believes, may even "modify greatly their endosmotic relations." He is inclined to think less than some other teachers do, of the importance of *chemical action* in therapeutic agencies.

"It is not impossible that the peculiar effects of medicines on the system may sometimes result from chemical reaction between the medicines and the tissues with which they are brought into contact while in the blood."

"This, I repeat, is possible; but it has not been proved positively that medicines do really produce their peculiar effects in this way in any one instance; and it is altogether premature to explain, as some are disposed to do, the whole agency of medicines upon this principle. The only well-ascertained fact which can be adduced in support of the hypothesis is that certain medicines, particularly the metallic, may be detected in the substance of the organs, often for a considerable time after they have been taken." (Vol. i. p. 20.)

"In relation to the process of alteration, it is highly probable that, in many instances, it is purely the result of chemical reactions set on foot by the remedy in the interior of the system; but we have little positive knowledge upon the subject, and theoretical speculations can lead to little practical good, except in so far as they may serve as a guide to inquiry and experiment. They should not be allowed to serve as the basis of curative methods, until the chemical reactions have been experimentally traced out, and demonstrated beyond reasonable doubt." (Vol. i. p. 56.)

Besides 1, Mechanical or Physical Methods; and 2, Chemical Methods; we have 3, *Physiological*, *Vital*, or *Dynamic* Methods, of primary operation of Medicines. The latter division is thus enunciated:

"Most medicines probably produce their peculiar effects by operating on the vital properties of the system, or of the part affected. Of the nature of this action we are quite ignorant, and must remain so until the nature of life itself is better understood. In the present state of our knowledge, all that we can say is, that the living structure is endowed with certain susceptibilities, and medicines with certain properties or powers, through which, when the two are brought together, certain changes of condition or action take place in the former. The medicine is said to act, and the living structure to be acted on, because it is in the latter that the changes are most obvious, and most interesting. This kind of operation is called physiological, or vital, in reference to the character of the effects produced in the system, and dynamic, in reference to to the supposed possession of an active power by the medicine. We may speculatively ascribe the results to physical or chemical reaction; but this explanation is, in the great majority of instances, destitute of the shadow of proof, and is indeed altogether insufficient, with our existing knowledge of physics and chemistry, to account for many of the phenomena. Until, therefore, further light is obtained, it is safest to be content with the mere statement of the fact." (Vol. i. p. 21.)

This view is still further elucidated in treating of the action of *alteratives:*

"They may change the condition of the blood, and may do so either chemically or dynamically; that is, in the former case, by taking something from, or adding something to that fluid through the influence of affinity, or by causing new reactions among its ingredients through the mere influence of presence, as emulsin, added to a watery solution of amygdalin, causes a reaction resulting in the generation of hydrocyanic acid; or, in the latter case, by operating on the vital susceptibilities of the living constituents of the blood, and changing it through modifications in the action of those living constituents. The chemical result might be produced equally in the blood removed from the body, and destitute of life; the dynamic results could happen only in the fluid while still living. Thus, alkalies may be supposed to render the fibrin of the blood more soluble, and in this way diminish its coagulability; while the presence of mercury in the circulation may bring about the same result by poisoning the fibrin, as it were, and affecting it through its vital properties." (Vol. ii. p. 229.)

The effects of medicines are, in another place, stated to consist in an alteration either of the organization or structure, or of the function or action of the body, or of some one or more of its parts; and, with some qualification of the strictness of the terms, a majority of medicines are considered to be exclusively functional in their operation. The "cell-action" of glands for instance, is believed to be capable of increase or diminution in its rate, by medicines, without alteration of structure. But, is not the "cell-action" identical, according to accepted views, with a change of the cell-substance, in almost every case?

The influence of quinia, opium, alcohol, ether, and other agents, is set down by Prof. Wood as *dynamic*, "that is, upon the vital properties of the parts affected, and not through any chemical combination with the tissues."

The use of the term dynamic, in this mode, is very convenient; the idea conveyed, excluding, as we have seen (in the quotation above, in regard to alteratives) *catalysis*, as well as ordinary chemical action, and applying it only to *vital* influences, is important; but, we wish it could be more clearly established. Here, as with Headland,* we are led, by the impossibility of accounting fully for the action of most medicines on mechanical or chemical principles, to *conclude* their influence to be *vital*, as distinguished from either of the two former. But, does this follow? Is there, in fact, any *sequitur*, as Dr. Wood has once or twice acknowledged, but that of *our nescience?* Still, the convenience of the term is such as to lead us, while it is not urged as a finality, to desire its provisional continuance.

In Part 1, Chap. III. we have an arrangement of the "Modes of Therapeutic action, or Therapeutic Processes." These are, 1. Depletion. 2. Repletion. 3. Dilution. 4. Elimination. 5. Stimulation. 6. Sedation. 7. Revulsion. 8. Supersession. 9. Alteration. 10. Anti-causation. 11. Chemical Action. 12. Mechanical Action.

By one of these terms, that indicating "the cure of a disease by the removal of its cause," the ear is not very agreeably impressed. Might not "opposition," or "counteraction," or "antidotal, or antagonistic action," have supplied the place?

All systematic medicines are stated (Chap. II.) to be either *stimulants*, *sedatives*, or *alteratives* to the functions. The classification of Prof. Wood given in Chap. IV. would seem to be based, although with very great improvements, upon that of Dr. J. Murray.

In it, astringents are classed as "Permanent Stimulants." We cannot but have some difficulty with this placing; although, perhaps, it may be viewed as having no great comparative importance. Are astringents *stimulants* at all? Of the class, as detailed by Prof. Wood, *cold* is said to be astringent "incidentally to its *sedative* effects." Galls, if tonic, have all of that property associated with *bitterness*, apart from their astringency.

* Action of Medicines, p. 59.

Tannic acid is not absorbed into the blood unchanged; and therefore is not a "general" remedy at all. Alum, within Dr. Wood's observation, is sedative at least to the nerves of taste, which it blunts perceptibly. Lead is treated of, explicitly, as *astringent* and *sedative.* On the whole, perhaps Headland's idea may be considered as, here, nearly correct; unless that too much reference is made, in proportion, to the *muscular* system as specially affected by astringents.

"I conceive," says he,* "the astringent powers of these substances to depend very much upon their chemical affinities." "Nearly all astringents have the power of coagulating or precipitating albumen. Tannic acid precipitates gelatine too." "They appear to constringe fibrinous as well as albuminous substances, even many dead animal matters, by a chemical action."

Under these circumstances, might they not be placed, rather among *sedatives*, or *local remedies*, than where they are?

Three kinds of astringent action at least would seem to be traceable: 1. Contraction of the organic muscular fibres, *e. g.* of the blood-vessels, as by cold; 2. Sedation to nervous organs, as by the preparations of lead; resulting in diminution of the circulation, &c., of the part; 3. Chemical constriction, as in the action of tannic acid upon the skin or other parts; to which we may add, the *styptic* influence of creasote and other agents, which coagulate the albumen of the blood or of various transudations.

The use of the phrase "*vital cohesion* of the living tissues" is, we think, liable to exception; although the omission of the adjective, vital, would have rendered it innocent. The mere fact, it seems to us, of the rounded form of all tissues, constructed under the agency of the life-force, would show that it is an *expansive* and *excentral* force, and thus, like heat, the antagonist of cohesion: as inorganic forms are crystalline, from centripetal attractions, and extraneous pressure; while the *cell* is a life-bubble, blown by a central power, overcoming physical, and modifying chemical resistance. This is saying, perhaps, a good deal about a word; but the phrase named is repeated in several places, and, in fact, involves a point of some consequence in the *physiology* of Therapeutics. Thus, as to the Mineral Tonics:

* Action of Medicines, p. 255, &c.

"Producing a slight increase of the vital cohesion of the molecules of the tissues, they give them greater power, while, by a gentle excitation, they call this power into a somewhat higher exercise. But, while thus generally tonic and astringent, they are disposed to act more especially on the nervous centres. . . . . . Here we have the secret of one of the most important therapeutic effects of this set of tonics; that, namely, of controlling the irritability of the nervous centres, and thus obviating various nervous disorders, and especially muscular spasm." (Vol. I. p. 387.)

In regard to Tonics, as a class, the following definitions and explanations are given:

"Tonics are medicines which moderately and somewhat durably exalt the vital actions." "It is necessary to discriminate between strength and action. The former is evidently the capacity to act, the latter the exercise of that capacity. By increasing the latter, we do not necessarily increase the former. Tonics do not, therefore, essentially augment strength, and the name of *roborantia,* or *corroborants,* by which it has been proposed to designate them, is not appropriate." (Vol. I. p. 175.)

It is, perhaps, an illustration of the difficulty of consistency in all classifications, upon complex topics, such as those of the Materia Medica, that we find, notwithstanding, (p. 183), Tonic Diet mentioned, as an agency calculated (we presume) to increase strength as well as action; as a tonic of animal origin, cod-liver oil, is named, which is considered by many as nutrient as well as medicinal; quinine is said to "probably augment the quantity of the blood, and to render it richer" (p. 241); and, lastly, *Reconstructive* Tonics are defined (p. 425), as "tonic substances which *enter essentially into the constitution of the system*;" chalybeates making up the whole of this section. Practically, also, Dr. Wood acknowledges, of course, that, when debility exists, "tonics may prove not only stimulant but positively strengthening, provided, &c." (p. 178).

Leaving these verbal and theoretical points, we may pause, for a moment, with advantage, over the views of the distinguished and experienced author, in regard to one or two of the most important of the tonic medicines. *Cod-liver oil* has been tested, in its therapeutic use, under the eye of Prof. Wood, in the wards of the Pennsylvania Hospital, as well as in the private practice, with the very best opportunities for judging. His decision, therefore, may be regarded as having the value of authority, however it may resemble the verdict of the majority of other observers. In regard to its mode of operating,

"It has been said that it fattens by simply supplying oil, in a state in which it will readily enter the blood, and that other oils of easy assimilation will answer the same purpose. But it does not simply fatten. It improves the digestive process, increases the proportion of red corpuscles in the blood, and invigorates the whole nutritive function. The mere increase in the proportion of fat in the system is one of the least important of its results. Besides, other fats do not produce the same effects. Nothing has been more common, in the Hospital of which I have charge in the winter season, than to see consumptive seamen rapidly improve in their condition, under the influence of the oil, though they may have been previously consuming, on shipboard, much larger quantities of oily matter in the shape of fat pork." "I think it must be admitted that cod-liver oil has positive medicinal properties; and the best explanation, I think, of its operation is, that it possesses the power of directly stimulating the blood-making and nutritive functions, in a manner analogous to that of other tonics, and, in certain cases, more effectively than they." (Vol. I, p. 203.)

It is now believed, by many physiologists, that the liver, in vertebrated animals, partakes in the function of *assimilation;* may not the fatty matter laid up in this organ, then, be prepared, so far, for taking part in cell-formation, as to be much more subservient to the nutritive processes, even of other animals, than any other fat, or almost any other material? Prof. Wood does not deny that the oil serves the purpose, to some extent, of nutriment. A patient of the writer of this notice lived for two weeks, and gained strength (while suffering from gastric irritation) upon no other food than a tablespoonful of cod-liver oil, taken every night on going to bed. It is important, as it has been remarked, that the term *cod-liver* oil should be retained; as, the oleaginous matter from no other part of the fish could be expected to have similar powers. We might anticipate the valuation placed upon this remedy by Dr. Wood, from what has been already said. It is esteemed by him as one of the most important articles of the Materia Medica; exerting an *extraordinary* influence over *scrofulosis*, whether external or tuberculous; and indicated generally in cases of chronic debility, with impoverished blood, and defective nutrition or assimilation, not connected with inflammation of the stomach. In regard to phthisis, he says (p. 210):

"Of this I have no doubt; that, if begun with early, and used perseveringly, with the aid of other measures calculated to invigorate the general health, it is capable of *very considerably diminishing the amount of mortality* from this fearful disease."

If, then, when the consumptive must be told that—

> "The fields for thee have no medicinal leaf,
> And the vexed ore no mineral of power,"

the *sea* yet gives up its hosts for his rescue, it should lead us to look farther into the same domain, for other treasures, to add resources to our art. Marine algæ have, already, by the hands of British and American caterers for the afflicted, been introduced as valuable substances of nutriment, and perhaps of alterative power.

Quinia, and its congeners, Cinchonia and Quinidia, are believed by Dr. Wood to produce, in small doses, effects like those of the simple bitters. In larger quantities (from 6 to 12 grains daily,) they tend to act specially upon the brain; not affecting the circulation to any important degree. When from 12 to 60 grains or more are given daily, in divided doses, the effect upon the cerebral functions is increased, and a decided sedative influence upon the circulation is produced, as shown by the pulse. The latter effect is considered to be secondary, and dependent altogether upon the former. The operation of a single dose, just large enough to make itself felt, say five or six grains, is said by Briquet to continue generally two or three hours; of double the quantity, given through the day, in divided doses, about eight or ten hours; of larger amounts, given in the same way, up to a drachm daily, from twelve to thirty-six hours. The writer of this article has been able to confirm, by repeated experiments upon his own person, the conclusions evolved by Briquet, in regard to the fact of the principal influence of quinine being *cerebro-nervous*, and as to the more or less prolonged period of its duration.

Dr. Wood prefers, for the anti-periodic effect of the sulphate of quinia its exhibition in *small doses, repeated at intervals of an hour or two.* As every one will have an opinion upon this subject, based upon supposed experience, we may state our conviction to be very decidedly in favor of the view taken by Dr. Wood, as opposed to the use of larger doses at longer intervals.

Many practitioners, however, will find a difficulty in maintaining the same confidence which Dr. W. enjoys in the "almost certainty" of quinine in "all regularly intermittent or periodic diseases." "I do not remember," says he, "to have met with a case which has not yielded to it, since the management of this medicine has been well understood."

That it will *interrupt* the course of Intermittent Fever, almost invariably, is certain. But, that a considerable number of cases, which have been allowed (with how much early neglect we know not) to become chronic, will not be cured by it, we are sure; notwithstanding the adoption of the plan recommended by Dr. Wood, of *anticipating* the weekly or bi-weekly period of its return, by *quininization*, the remedy being suspended in the interval. An experience, more positive than extensive, upon this point, has been fully confirmed by accounts given by several of those who practice in the midst of miasmatic districts. *Iron* is, in such cases, as we believe, a vastly better anti-periodic than any preparation of bark; and we have known *cod-liver oil* to answer the same purpose very promptly. Even with greater frequency, have we seen the failure of quinine in the most regularly intermitting neuralgia; and this when no organic disease whatever could be discovered to account for its persistence. One such case at least, of remarkable obstinacy (involving the eye alone, with a three weeks' interval), is upon record, as occurring in the wards of the Pennsylvania Hospital.

In regard to the *nature* of the anti-periodic action of the alkaloids of Peruvian bark, Prof. Wood denies the validity of the opinion which ascribes it to the property of "neutralizing the miasmatic poison" in the system; explaining it, on the contrary, by the "powerful influence exercised by the remedy upon the nervous centres, through which, probably, the paroxysms are produced."

No favor is shown by our author to the employment of quinia in large doses in typhoid fever; nor has he had any experience, or hopeful expectation, of the success of the practice of Dr. Dundas, in typhus, which has been so fairly tested abroad.

Dr. Wood takes that view of the composition of quinia, which allows the term neutral sulphate, applied to the ordinary salt, to be chemically correct. The dose of the sulphate of *cinchonia* is stated as one-quarter or one-third larger than that of the salt of quinia; while that of *quinoidia* (amorphous quinia) and of the sulphate of *quinidia* is about the same.

Passing to the section upon *diffusible stimulants*, we find, as those first considered, being the most general in their action, *heat* and *electricity*. The author expresses his indebtedness upon the latter subject, to M. Duchenne de Boulogne; "who, by his thorough and laborious experimental investigation of electricity in its medical relations, the sagacity with which he has traced the various ramifications of its influence, and the ingenuity and perseverance with which he has applied the knowledge thus obtained to successful therapeutic results, has given a precision to the subject which it before much wanted, and has opened a new era in the history of the remedy." Very full descriptions, with diagrams, are quoted, of the "Appareil magneto-faradique" and "Appareil volta-faradique à double courant," of this distinguished author.

The following is among the most important of the deductions established by M. Duchenne from his large experience.

"Great consequence has been attached to the transmission of the electric current along the nerve, and in one direction rather than another in imitation of the course of nervous influence. But much of what has been said upon these points has been purely theoretical. M. Duchenne has come to the following conclusion: In man, whatever may be the direction of the currents, or the degree of vitality of the nerves they traverse, the same results are always produced, when the conductors are applied to any portion of the course of the nerves; namely, muscular contractions and sensations." "Changes in the direction of the current exercise no appreciable influence over the muscular contractility or sensibility in man."

Upon the basis of these and other investigations, Prof. Wood considers electricity, so far as yet understood, simply as a *universal stimulant*, with a special excitant influence upon sensation and muscular contraction; all of its well-ascertained therapeutic uses being referable to these properties.

Alcohol, in the treatise before us, is located among the *cerebral stimulants*. Its operation of this kind, is said to be "probably dynamic." And yet, although not acknowledging the correctness of the opinion of Dr. Böcker, that it "retards the metamorphosis of tissue," Dr. W. states that

"It is an undoubted fact that the habitual use of alcohol lessens the desire and apparent necessity for food." "I am among those who believe that it may, through the agency of the vital forces, and in the presence of organized nitrogenous matter, be converted into any one or all of the proximate constituents of the body, excepting only the mineral. The one, however, which most obviously results, is oil; and this is often generated with great rapidity. It is not only visible in the increase of the adipose tissue, and in the promotion of obesity in certain individuals, but it exists also in abnormal proportion in the blood." "It is food, therefore, as well as a stimulant. But this fact in no degree justifies its abuse." (Vol. I. p. 665.)

These statements obviously confirm, in the main, the practical views of Dr. Chambers (on Digestion, &c.,) an analysis of which was given in the November number of the Examiner. Those views have been, and will be, opposed; as the subject is one especially open to controversy. Space for its discussion is not left to us in this article; but we cannot overlook, altogether, some points. How, for example, observes Dr. Wood, can an agent whose function it is to increase the activity of the brain and stomach, and other organs, at the same time, diminish the rate of disintegration of the tissues? We might answer, that there are three kinds of metamorphosis, involving the tissues, or the blood, or both. 1. *Constructive;* as the making of fat from sugar or from alcohol. 2. That of passive *organic decay.* 3. Of the *action* of musculo-nervous organs.

Now, the first of these gives *heat*—as Liebig distinctly states, in regard to the formation of fat in the body; oxidation being a part of it. Analogy confirms this, strikingly, as to *formative processes evolving heat,* in the rise of temperature well known to be produced in plants, at times of *germination and flowering.* But *heat* is *power;* Joule, Tomson, and Bernard have proved this without, and physiologists have shown it to be true within the body. One grand object of the evolution of heat in the body undoubtedly is, the production of power. And it is not necessary, we may well believe, for the process of change which produces heat (and power) always to occur in the substance of the tissue concerned. It may occur in the blood in contact with it. The general statement is,* whether universally true or not, that, "respiratory or calorifacient food is *burned off* by oxygen, without entering the tissues." The second mode of metamorphosis, *decay,* may produce heat, although not always availably. A gangrenous foot contributes little or nothing to the caloric or

* Liebig, Animal Chemistry; and Carpenter, Prize Essay on Alcohol, Am. ed. p. 100.

force of the body. The *third* mode is variable, with the amount of exercise we use. Medicine or food has little to do with this directly.

But, in regard to the two former modes of metamorphosis, material or "dynamic" agents may exert an influence. The daily amount of organic decay, in our common life, is probably mostly in excess. All that depresses vitality, increases it; and *vice versa.* Dr. Böcker has proved that (as shown by the excretions) it is *increased during sleep.** Virchow and E. Parkes have ascertained the same to be true of *fever.*†

By increasing the tension of the nervous system, then, it is probable that tea, coffee, tobacco, arsenic, coca,‡ opium, alcohol, retard, to its minimum rate, the passive disintegration of the body. We might venture, almost, to distinguish between functional and vital excitants. A functional excitant may be one which, by increasing action, gives aid to the *chemical* affinities of organs, and urges their *active* disintegration. A vital stimulant, on the contrary, may, by favoring the constructive processes, aid the vital force in maintaining its control, make the same amount of substance and force go farther, and economize both material and power. Largely given, by disturbing the organs, alcohol probably acts in the former capacity: in small doses and in appropriate conditions, in the latter.§ It is, as we believe,

* Brit. and For. Medico-Chirurg. Rev., Jan. 1856, p. 233.

† *Ibid*, April, 1856.

‡ Johnston, Chem. of Comm. Life, vol. ii. p. 121.

§ As reasons why alcohol might be expected to be an eminently heat-making "accessory food," besides its small proportion of oxygen, it should be recollected—1. That *hydrogen*, which is in excess in it gives out more heat in oxidation than any other substance; 2. That the *capacity for heat* of a mixture of alcohol and water is lower than the mean of the two liquids; so that heat is evolved by the mere entrance of alcohol into the blood; and 3. That as alcohol is *absorbed without any digestive preparation*, a considerable amount of force muscular and vito-chemical, is thus saved, which would be required for the gastric and intestinal (and masticatory) preparation of more resistant and insoluble substances. It need not be doubted, moreover, that the physical change produced by alcohol in the animal structures, described as *corrugation* by Dr. Carpenter, (Prize Essay, p. 25,) and as "resembling the effects of astringents" by Dr. Wood (Therapeutics, vol. i. p. 649), may have an antiseptic value, and retard the metamorphosis of tissue. Supposing this to be especially true and important in tissues exposed most particularly to *air*, *water*, and *heat*, we may see how *the lungs* may be sometimes protected by it, against the disorganizing tendencies of the tubercular diathesis.

an opinion of much consequence, clearly put forth by Prof. Wood (Vol. I. p. 658, &c.), that alcohol does good (in cases requiring it,) *only in such doses as do not disturb the brain and other organs at the time.* It is a safe organic, but a dangerous motor and sensory stimulant.

One word more upon the theory of stimulation; even at the risk of our appearing somewhat *de trop* upon the subject.

Stimulation is defined by Dr. Wood as "the exaltation of any or of all the vital functions above the state in which they may happen to exist, at the time when the stimulating measures are resorted to." It is then said, that "a property common to all stimulation is that it is followed, in the ordinary state of the system, by a degree of depression bearing some proportion to the previous excitement." (Vol. I. p. 48.) Again: "One of the laws of all stimulation, whatever may be its degree, is that it is followed by a depression proportionate, at least approximately, to the previous exaltation of the function or functions excited."* (Vol. I. p. 478.) The same principle is asserted or implied by Dr. Carpenter, in his Prize Essay upon alcohol;* with an apparent recognition of the same definition.

Now, as to the universality of the truth of the above proposition,—with every desire to avoid paradox,—we cannot yet give assent. The authors we quote from do not seem, at least, to have given sufficient prominence to the necessary qualification. The nature of this can be seen by comparing a passage from the latter part of Dr. Carpenter's Essay,‡ with the sentences above cited.

He has labored to prove that alcohol is a *stimulus*, not a food; but now, in reference to certain therapeutical applications of it, he states that "in the cases in which alcohol is thus useful, there is an an *entire absence of stimulating effects.*" How can we reconcile these, with other familiar asseverations upon the same topic, without the perception that a *double* or *triple meaning* is unconsciously applied to the word "stimulation," and "stimulus." Stimulation is in one place the *excitation* of the body or some of its functions *above par.* In another, it is the *use of an agent* which, under certain circumstances, is *capable* of producing such an effect.

* See same volume, also, pp. 180 and 484.

‡ P. 165.

Dr. Carpenter acknowledges, indirectly, and Dr. Wood directly implies, an assent to the real law, which we believe to exist; namely,—that all stimulation which is *excessive*, is followed by a corresponding depression: while all that merely excites any function *up to par*, does, so far, only good, with no *resulting* debilitation—however it may fail, from want of other conditions, to sustain the organ or system at the point desired. To deny this, would be to ignore some of the most obvious and important of physiological data; which show that the body stands in constant relation to dynamic agencies, whose influence can be only considered as stimulant. Heat is a stimulant to the life-force; oxygen to all the active functions; blood is an excitant as well as food to all the tissues it reaches; and all those impressions upon the exterior of the body which give rise to instinctive and automatic actions, are stimulant, without any necessary ulterior depression. Now, in regard to alcohol, we believe that the evils,—all the evils, justly ascribed to it,—belong to the excess in which it is used. Without employing it out of proportion to the wants of the economy, but only as an "accessory" food, and in quantities not disturbant of the system at the time, we believe that no tendency to excessive thirst for it, no Circean spell or *oinomania* need be expected; unless in those sad cases where a hereditary proclivity exists, toward this, as toward any other disease.

We must pass from this exciting subject, however, and take leave of the work before us, with a brief reference to one more (among many) of the topics, the expression upon which, by such an authority, is of particular interest at this time.

We quote, with satisfaction, the following paragraph, in regard to the *abstraction of blood* as a remedy.

"There is no remedy more important than this; perhaps none which so frequently saves life. That it is susceptible of abuse, and often has been greatly abused, there can be no doubt; but this is only an argument in favor of a careful study of its powers for good and evil, and of great watchfulness in its use; none for its abandonment altogether; and the practitioners who reject it, and oppose their own prejudices and fears against the experience of all ages, not only deprive themselves of a most important agent for good, but assume a responsibility which might well make a conscientious individual shudder. *Though never one of those who might be considered as special advocates of the lancet, I fear, that in the reaction from an indiscriminate, and sometimes reck-*

*less use of it, the profession is now in danger of erring in an opposite direction; and cannot but think that the general tendency is rather to an injurious neglect, than to an injudicious use of the remedy.* In my experience as a hospital physician, I see many patients past all hopes of cure, whom early and judicious bleeding would probably have saved; but very seldom one, in whom I have reason to think that an abuse of the remedy has been productive of serious injury." (Vol. II. p. 37.)

The italics are ours;—and we believe the passage to be worthy of all emphasis; in fact, to be one of the most important of the teachings of these volumes. The more so, because to linger too long in the footsteps of the past, is not a characteristic of the author. Nor is he, on the other hand, in haste to abandon the lessons of experience for new dogmas. He well knows how to "conserve progressively, and progress conservatively;" and such minds always make the best guides and safest teachers.

We can only notice, farther, the introduction, into this treatise, in appropriate places, of the new orders, or subdivisicns, of *Spinal Stimulants* (Strychnia, &c.), and of *Utero-motor Stimulants* (Ergot, Cannabis and Gossypium); and the placing in the class of *Alteratives* of some substances not always so included; the whole list, with Dr. Wood, now comprising *Mercury*, *Arsenic*, *Iodine*, *Chlorine*, *Sulphur*, *Colchicum*, *Sarsaparilla*, *Guiacum*, *Mezereon*, *Sassafras* and *Dulcamara;* the modes of action of which are, of course, very various, although all are best comprehended under the term above quoted.

In a word, in making the survey of the *armamentaria* of the Art of Healing, as displayed in this admirable work,—to which we have done the most meager justice in this notice,—we meet with a great deal to encourage as well as to instruct. It may be believed, that the complaint, so often repeated, of the delinquent state of the science of Therapeutics, as compared with those of Pathology and Diagnosis, is due more to a want of knowledge *how to use* and apply the weapons it affords, than to their imperfection or want of number; and, perhaps, also, to a want of appreciation of the proper limits of possibility in Medicine.

In the beautiful Dedication to Prof. Franklin Bache, with which the Treatise opens, there is matter of interest, other, and perhaps higher than the scientific. In the mutual courtesy and co-labor of gentlemen connected, as Profs. Wood and Bache have so long been, with rival interests, we find much to admire. It does honor to our profession; whose differences have been, in the past, too much a by-word. The tribute of such a dedication as the one to which we allude, "is *twice* blest; it blesseth him that gives, and him that takes."

---

*The History and Statistics of Ovariotomy, and the Circumstances under which the Operation may be regarded as Safe and Expedient: Being a Dissertation, to which the Prize of the Massachusetts Medical Society was awarded, May*, 1856. By George H. Lyman, M. D. Boston: Printed by John Wilson & Son, 22 School street. 1856. Pp. 146.

We have read with considerable care and interest the above dissertation by Dr. Lyman. It is obviously the result of much patient and laborious research, and fully justifies, both by its style and matter, the award rendered it by the Massachusetts Medical Society. Its table, which comprises three hundred cases (a larger number than were ever brought together by any previous writer,) is drawn up with great care and accuracy, the original authority appearing to have been referred to in every instance. In fine, without any further comments, we pronounce it to be the best, as it is the fullest essay ever written upon the subject.

The following are some of the facts gathered by the author:—

"In three-tenths of the cases, the operation could not be completed.

The rate of mortality in all the operations was 40·13 per cent.

In seven-tenths of the cases, the operation was completed, with a resulting mortality of 42·78 per cent.

In the unfinished operations, the mortality was 30·68 per cent.

The proportion between the whole number of recoveries, *after the removal of the tumor*, and the whole number of operations undertaken in hope of such a result, we find to be as 39·66 to 100, or less than two-fifths!

Adhesions caused the abandonment of the operation in 22·06 per cent. of the whole number, or caused 77·27 per cent. of the failures.

No tumor was found in nearly three per cent. of the whole.

Where adhesions complicated the removal, 47·82 per cent. died; where no adhesions complicated the removal, 32 per cent. only died.

Of the whole number of short incisions, 30·76 per cent. died; of those completed, 38·33 per cent. died; of those not completed, 22·80 per cent. only died.

Of the whole number of long incisions, 41·95 per cent. died; of those completed, 41·46 died; and of those not completed, 45 per cent. died.

Previous tapping does not always cause adhesions.

As far as these cases go, the mortality is least between the ages of fifty and sixty, and greatest under twenty.

The mortality is least when the disease is of between three and four years' duration.

There is but little difference in the mortality between the married and single.

The right ovary is more often diseased than the left, though less so than often stated.

Of the above fatal cases, 42·35 per cent. were from peritonitis, 23·52 per cent. from hemorrhage.

Death ensued, upon an average, the eighth day; the average of deaths from peritonitis being also the eighth day; and those from hemorrhage in twenty-two hours.

And, finally, in more than ten per cent. of the cases, important errors of diagnosis occurred."

The following summary will afford our readers the conclusions at which the author has arrived:—

"1. The mortality attendant upon Ovariotomy is no greater than it is after other capital operations.

2. The mortality resulting from extensive incisions of the peritoneum is generally over-estimated.

3. Fully developed cystic disease of the ovarium tends rapidly to a fatal result.

4. No method of treatment heretofore devised for it is so successful as extirpation; excepting, possibly, that by injection with iodine, of the results from which we have, as yet, insufficient statistics.

5. The operation is unjustifiable in the early stages of the disease.

6. After active development has commenced, with the supervention of constitutional symptoms, the sooner the operation is performed, the greater the chance of recovery.

7. No rule can be laid down as to the length of the incision, other than the general one,—that the shorter it is, the less the mortality; and that, therefore, the primary incision should always be small and extended afterwards as may be necessary, according to the exigencies of each particular case.

8. If, after the operation is commenced, extensive adhesions should be discovered, either the complete abandonment of the intended extirpation, or the attempt to cause suppuration, and gradual contraction of the cyst, by means of a permanent external opening, are to be preferred to the division of the adhesions, and completion of the operation as originally designed."

Our readers will perceive that the mortality (40.13 per ct.) resulting from the operation is much higher than was previously supposed. Dr. Atlee makes it only 26½ per cent.; this is done, says Dr. Lyman,

"By throwing out of the calculation twenty-seven cases which were complicated with other diseases, six cases in which accidental occurrences were supposed to be the cause of death, and three cases in which death did not ensue for some time after the operation. Now, these same complications are just as likely to be met with, in the same frequency, in all future operations, unless the differential diagnosis of ovarian disease is greatly improved."

High as these statistics show the mortality to be, we are satisfied it is much lower than the reality. Many unsuccessful cases never meet the light. Under the heads of "anonymous" and "unknown," fourteen cases, for instance, obtained from various sources, are recorded, all of which were fatal but one, and in that one no tumor was found. In two of these instances the operators were unwilling to give either the details or permission to publish their names. We have no doubt, also, that many cases published as successful by operators, are incorrectly so given. In his remarks before the London Medical Society, (April 5th, 1854,) Mr. Pilcher stated, "that in the practice of a gentleman eminent for this operation, he had heard of three cases which had been regarded as successful, but which had proved fatal from the effects of the operation." Dr. Ashwell, in his Treatise on the Diseases Peculiar to Women, makes a somewhat similar statement:—

"In these remarks, (alluding to a statement of Mr. Safford Lee,) I certainly concur, especially as to the ultimate and in most instances early mortality of many of the patients said to have recovered. My own enquiries have furnished me with some painful knowledge of this kind."

But this is not all. In 88 cases—three-tenths of the whole number—the operation was abandoned or only partially completed. Of this number, in 8 of which no tumor was found, 27 cases or 30·68 in 100 died. What became of the remaining cases? They escaped, we know, the immediate death that fell to the lot of the 27, but what was their condition and how long did they live afterwards? On these points but little information is afforded us. It is mentioned of some of them, it is true, that they died a few weeks, six months, two, three and five years afterwards; of some that they were living several years afterwards; several were tapped, one seventeen times, several had children and one lived twenty-five years after the operation. Of the condition and duration of life, however, of the majority, we are left in entire darkness. Information on these points, also, would aid us greatly in forming our opinion of the necessity of the operation.

The author compares the mortality of ovariotomy with that of other capital operations, and does not deem the comparison discouraging. To the objection, that in most capital operations the alternative is death, he remarks :—

"This assertion is doubtless true of many of the traumatic cases in which amputation or ligature of large arteries is performed, and hernia also; but ligature for aneurism, amputation for necrosis, articular disease, or other pathological states, derive no advantage from the argument; for they are certainly as amenable to medical treatment as are those cases of ovarian disease which have usually been operated on."

Undoubtedly they are, perhaps even more so. But this does not seem to us to be the point at issue. The question is, whether death is as equally inevitable from ovarian disease as it is from aneurism, necrosis, &c. To this we think there can be but one answer—that it is not. Numerous cases of disappearance of the tumor (by spontaneous rupture, inflammation of the sac, &c.,) are recorded by writers, besides which, it is by no means uncommon to find the disease remain dormant for years. We are fully satisfied that in many of the cases that have been operated on, there did not exist the same urgency for the knife as there does in the other pathological conditions cited. We have no doubt, in fact, that many of them were similar to the following related by Dr. Ashwell :—

"A young woman, under 22, had ovarian dropsy; her countenance bespeaking excellent health, and her history confirming the impression. Without interference, many years might have been added to her existence; and, as one of the fortunate incidents of life, it might have so happened that the tumor should cease to grow. But unhappily she was convinced that extirpation was proper; the operation was most ably performed, and in a few days she died."

It should be remembered, also, of ovarian disease, that the operation cannot be completed in three-tenths of the cases; a condition of things which renders it dissimilar to all other operations. The mortality of those unfinished cases, too, is nearly 31 per cent., the remaining cases being left in no better condition, generally, than they were in previously.

The difficulties of diagnosis, also, are so great, that in more than ten per cent. of all the cases reported, important errors occurred. In several instances, no tumor even, "only flatulence and fat," existed. We make these remarks to show how impossible it is to draw any comparison between it and other operations. There are circumstances and considerations attending it which render it unlike any of these. It stands alone, and must be judged of by itself.

Of the 300 cases collected by Dr. Lyman, 23 were performed by Dr. W. L. Atlee; 32 by Dr. F. Bird, and 50 by Mr. Clay—105 cases by three operators—one-third of all the cases on record! Of Dr. Atlee's cases, the operation was completed in 19; in 4 not completed on account of adhesions, &c.; of the complete operations 11 died or 58 per cent.; one case tabled as a recovery died 4 months afterwards of erysipelas; of the 4 incomplete cases 1 died. Of Dr. Bird's cases 12 were complete operations, and 20 incomplete; of the complete 4 died, or 33 per cent. Of Mr. Clay's cases 40 were complete and 9 incomplete operations; of the complete 14 died, or 34 per cent.; of the incomplete 2 died.

In the list of operators we find, with some exceptions, very few distinguished names. It is a singular circumstance that in our own city, save in one instance, the operation seems never to have been performed but by Dr. Atlee.

---

*A Treatise on the Practice of Surgery.* By Henry H. Smith, M. D., Professor of the Principles and Practice of Surgery in the University of Pennsylvania, etc. etc. *Illustrated by two hundred and seventy-four engravings on wood.* Philadelphia: J. B. Lippincott & Co. 1856. 8vo. pp. 828.

The "critical" reader of this work is notified, in a deprecatory paragraph of its preface, that it was "written during hours taken from repose and with the frequent interruptions of active professional life." Few similar works, we suspect, of really practical value, are or can be prepared under different circumstances. It is, unhappily, the common and inevitable lot of professional authorship to be thus hampered and impeded in the discharge of its responsible duties. Embarrassments of this kind are to be anticipated and provided for, and hence we regret to meet such a plea in excuse of any evidence whatever, whether "typographical" or "otherwise," of hasty composition in a volume especially addressed to students, and by one whom they are to look up to and follow as their master in the branch which it discusses.

Still, we desire to appreciate fairly both the difficulties of the task and the self-sacrificing industry suggested by our author's caveat in his own behalf; and were we to obey our inclination only, we should certainly take shelter under the same ancient apology and close the present notice with two brief remarks. We should content ourselves with saying, in the first place, that the new text-book on Surgery was handsomely illustrated and got up, and that it was otherwise as good as could be expected under the circumstances of its preparation; in the second place, we should cheerfully express the hope that the author might be speedily enabled to fulfil his promise of improvement in a future edition.

Were Dr. Smith's book the comparatively irresponsible production of a private teacher merely, and were it offered as a primary and elementary manual only, we should gladly escape from further discussion of its character and claims upon our readers' notice. But it is issued by the Professor of Surgery in the University of Pennsylvania as one of the text-books, or a portion of the text-book, to be used by the members of the class in attendance on his regular course of lectures. It is entitled a "Treatise on the Practice of Surgery," and is offered as a "Text-book," to serve as an "adjuvant" to the author's "lectures on the Principles and Practice of Surgery," which he has endeavored so to arrange that "whilst it will recall many of the opinions taught in his course, it will also facilitate the progress of the young surgeon, and prove useful to him as a work of reference in the responsibilities of early professional life." The preface further informs us, that "although the title of Practice of Surgery has been taken, the work will be found to contain as full an exposition of the principles of the science as was consistent with its character as a text-book."

Again, "In the composition of this Treatise, it has been the author's wish to present each subject as fully as was essential to its comprehension by the youngest of his pupils, without entering into such details of the history, pathology, &c. of each as properly belong to monographs. Such an extended treatise on each of the various affections that are daily presented to the attention of the surgeon, would have enlarged this work beyond reasonable limits, and destroyed the object of its formation. Such reading should also be made to occupy the years which ensue upon graduation, and is amply provided for in the works of those who have devoted themselves to special subjects, as in the Treatises on Fractures, Luxations, Diseases of the Breast, Testicle, Hernia, &c., by Sir Astley Cooper; The Affections of the Bones by Stanley; of the Eye by Lawrence; of the Ear by Wilde, &c. But to all these subjects sufficient insight is now offered to tempt the student to further investigations when he has mastered the elements of his profession, if the love of knowledge in him, as in others, grows with its cultivation."

We have been careful to quote the language of the preface, in order to avoid misrepresenting the author's idea of the aims of his Treatise or Text-book, as it is indiscriminately called, and of its claims upon the attention and confidence of the student and the young practitioner. According to this language we may presume that he shrinks from none of the responsibilities involved in the double office of author and professor, and that he is ready to be weighed in the balance which is demanded alike by his position and pretensions as a teacher.

Tried by such a test, we fear that his recent essay will be found most lamentably wanting. We are constrained to say this, in strict justice, after a careful examination of the volume. We should be glad indeed to welcome a genuine American book on surgery, provided it were a sufficient substitute for the re-prints with which the country has been flooded; but we cannot regard the one before us as even a tolerable succedaneum for its predecessor in the same school, while we are bound to admit that it is not likely to supersede, either as an epitome, text-book or systematic treatise, the one volume compends of Pirrie, Druitt and Erichsen.

We are no advocates of the multum in parvo style of circulatting practical instruction, nor have we the least desire to recommend any of the popular compressors of surgical knowledge, in place of more elaborate works, even to the tyro in medical pursuits; but we do know that all such sparklings of the true lights of science are eagerly sought for as almost the only available sources of illumination to the majority of the students of our country, at least until the attainment of the medical degree and the needs of actual practice allow and demand a more extended range of reading. The best, therefore, that can be done for the pupil of the class-room, is to supply him with the most reliable and digestible amount of didactic nutriment which can be found for him within the compass and in the portable form to which he insists upon confining it—to give him such mental food as he consents to swallow, and as may eventually and safely lead him to a taste and aptitude for better things.

Looking upon Dr. Smith's octavo as intended to supply this limited want alone, we cannot help being struck with many faults of omission and commission, which seriously impair its value, even as a memento of the professor's daily lesson, and which render it still more unsatisfactory as an aid to the junior practitioner.

That our readers may judge in some measure for themselves, let us take a general survey of the arrangement and contents, and look a little into his mode of treating certain of his topics.

At the very first glance the reader will encounter a sort of congenital deficiency, which, although not seriously crippling in its effects, is yet at least unfortunate in a school book intended partly for beginners. We allude to the hiatus in the table of contents, nearly the whole of the latter half of which is not to be found, all after page 496 having been left out. Happily, however, this discouraging accident is not the forerunner of similar short-comings in the subsequent page. On the contrary, there is abundant reason to be pleased with the manner in which the proof-reader has discharged his trust, as well as with the general typographical appearance of the text and illustrations.

The only complaint we wish to make in this relation concerns the author rather than his assistants, and refers to a habit of mis-spelling well-known names, which is inexcusable under any press of occupation, above all in a professor. Thus, is the advanced student annoyed and the junior one misled with the continual substitution of Dessault for Desault, McCartney for Macartney, and Hines for Hind, whenever they are quoted!

The work is divided into seventeen parts. The first three of these appear to be occupied with "surgical disorders in all parts of the body;" the next two are devoted to "those which affect the different tissues," and the remainder (from VI to XVII) include

"the consideration of such as are specially related to certain regions of the body."

Part I. is headed "Surgical Pathology and Therapeutics." It is occupied with brief and occasionally confused hints concerning surgical diagnosis, but contains nothing whatever upon therapeutics. "Surgical Pathology of the Soft Tissues" is the caption to Part II. This part includes the study of inflammation in its "General Characters," its "Etiology and Treatment," its "Effects or Products." Under the same head we have also "Abcesses," "Hectic Fever and Pyemia," "Ulceration and Ulcers," "Mortification," "Specific Forms of Inflammation" (furuncle and anthrax), "Burns," "Effects of Cold," "Erysipelas." The "Pathology of Abnormal Growths in the Soft Tissues" is the title of Part III. It includes, in two chapters respectively, the study of "Malignant Deposits or Growths" and of "Benignant Tumors."

Under the head of "Injuries of the Soft Tissues," we have in Part IV a chapter on the "General Character of Wounds," one on "Special Wounds," another on "Gunshot Wounds," a fourth on "Tetanus," and a fifth on "Wounds of the Regions of the Body." Next we have Part V, devoted to the "Injuries and Diseases of the Bones," comprehending, of course, the different fractures. Then follow Part VI, on "Injuries and Diseases of the Joints;" Part VII, on "Affections of the Eyeball and its Appendages;" Part VIII, on "Diseases of the Ear;" Part IX, on "Affections of the Nose and its Cavities," and Part X, on "Affections of the Throat and Neck."

Part XI, on "Affections of the Abdomen" contains but one chapter of some fifteen pages, and that is occupied entirely with "Hernia." Part XII treats, according to its heading, of "Diseases of the Genital Organs," under which are classed venereal diseases and their sequelæ, and spermatorrhœa.

"Stone and Gravel" afford the subjects of Part XIII, under the title of "Affections of the Kidneys and Bladder." "Affections of the Testicle and Cord" furnish the topics for Part XIV; "Affections of the Anus and Rectum" those for Part XV, and "Affections of the Blood Vessels" (including the different forms and kinds of aneurism only), those of Part XVI. Part XVII

on "Affections of the Extremities" terminates the volume in two chapters, the first of which treats of "Paronychia," "Enlarged Bursæ," "Ganglion," "Housemaid's Knee," "Femoral Bursæ," Neuralgia (!) and "Varicose Veins;" and the second is occupied entirely with the different varieties of club-foot.

We have, in thus enumerating the different leading heads as given by Dr. Smith, together with those of a few of the more prominent chapters, endeavored to present some idea of the classification as well as range of topics in his "Practice of Surgery."

The array of material thus briefly sketched, although far from displaying the logical connection, order or completeness expected in a systematic treatise, would seem at a cursory view to be tolerably comprehensive for elementary purposes, sufficiently so, perhaps, for those of an "adjuvant" to the author's course of lectures. But a closer inspection of the pages of his work unfortunately weakens these impressions in its favor even as a syllabus. The most inexperienced reader must be struck with the unequal amount of attention given to many subjects in proportion to their respective importance; and more advanced students cannot fail to be equally surprised at the absence of some topics altogether, as well as at the presence of others which are seldom met with in a volume which is too limited in space for much that ought to have a place in this. We find, for instance, that although (for no valid reason except the personal one of the pre-existence of a separate work upon it by the same author,) operative surgery—all the high art of the branch—is unceremoniously cut off with a barren reference wherever it should have been presented, minor surgery with its attendant common places of venesection, leeching, cupping, bandaging, poultices and other matters of simplest routine, are made to occupy the post of honor in whole chapters of the book! The humblest duties of the dresser, which every beginner should learn in his preceptor's office, are dwelt upon and illustrated with a tedious prodigality of detail. The marginal engravings are well executed throughout the book; they do great credit to artist, printer and publisher; but we must confess that some of those which are "after nature" impress us as remarkable specimens of original design. Our humble opinion is, that the majority of these grand effigies of slings, handkerchiefs, bandages and—poultices (!) are decidedly more interesting and useful in the author's duodecimo on Minor Surgery. They might advantageously give up the large space they occupy in their new position, to a great deal of genuine theory and practice which has been sadly hurried over in filling up the sections on the treatment of inflammation, of wounds, and of various other injuries and surgical disorders.

Admitting for the moment, however, that minor surgery may thus take precedence, and to the entire exclusion of blood-shedding operations, in a treatise on the practice of surgery, we are bound to insist on the only plausible substitute,—a full share of the "Principles of Surgery"—in a word, as the author himself proposes, a body of the "science" to form an adequate accompaniment to the "treatise" on the "art" already published; "the two works being intended to form one series on the Science and Art of Surgery." Now we strongly suspect that few intelligent members of the class in surgery will be content with the amount of surgical philosophy embodied in the work before us, or with the vague and superficial manner in which precepts and doctrines of grave moment are conveyed in respect to many questions which belong to the simplest course upon the branch.

An unexpected curtailment of our space obliges us to close with very few of the references which we had intended to present, in justification of the foregoing remarks. In his treating of wounds we no where find anything about the *shock*, the prostration or collapse of injury. It is barely alluded to in gun-shot wounds, and then only to underrate its gravity. It is ignored in railroad injuries, injuries of the head, of the abdominal viscera, compound fractures, burns. Eleven pages are occupied with hemorrhage, and seventeen with poisoned wounds; ten pages are devoted to gun-shot wounds, and ten to wounds of the head. Wounds of the face are despatched in two and a half, and those of the neck in one and a half! Two pages suffice for wounds of the chest, including two lines on wounds of the axilla! One page is deemed enough for wounds of the abdomen, and one for those of the intestines, while two and three-quarter lines finish wounds of the kidney, and about the same suffices for those of the bladder! Half a page is appropriated to injuries of the genito-urinary organs. Of this, ten lines are employed with wounds of the buttock and perineum, the treatment for which is cleansing the parts and applying a poultice, to be retained with a handkerchief!!

It further appears that three pages are given to sprains, and three and three-quarters to arthritis. Poisons in the stomach furnish matter for five pages; seven lines are vouchsafed to tertiary syphilis, and seven pages to spermatorrhœa! Four and a half are enough for hydrocele, while over ten are required for tubercles of the testis!

In addition to meagreness in essentials and redundance in their opposites, and other drawbacks already noted, we might easily show a lack of clearness and precision of ideas as well as language, which is particularly obnoxious in a student's guide. We have said quite enough, however, to acquit ourselves of an unavoidable responsibility and most unwelcome task—the hardest of its kind that has ever fallen to our lot. With the best feeling towards our author, and the warmest attachment to his school, we are none the less manifestly bound by our obligations to nothing extenuate, while we set down naught in malice.

---

*The Obstetric Memoirs and Contributions of* James Y. Simpson, M. D., F. R. S. E., &c. &c. Edited by W. O. Priestly, M. D., Edinburgh, &c., and Horatio R. Storer, M. D., Boston, U. S., &c. Volume 2nd. Philadelphia: J. B. Lippincott & Co. 1856.

As the prefatory note informs us, this present volume contains a great variety of essays and contributions, some of which have never before been published, while many of our old acquaintances have been lengthened and elaborated.

The heads of the subjects treated of are as follows:

The Pathology of the Puerperal State.

The Physiology and Pathology of the Products of Conception,

The Pathology of Infancy and Childhood, and

Anæsthesia.

The contributions which appear for the first time in this volume are most interesting, and will amply repay an attentive perusal, particularly the Memoir (page 46) entitled "Pathological Researches on Puerperal Arterial Obstruction and Inflammation," and the essays "On the Rudimentary Reproduction of Extremities after their Spontaneous Amputation, (page 344.)

There are likewise several valuable novelties on the subject of local Anæsthesia. Indeed, we can safely say that this volume is altogether one of the most interesting medical collections we have ever opened.

About one third of the book is entirely devoted to the subject of Anasthesia by Chloroform, which is preferred by Dr. Simpson as an anæsthetic agent to the sulphuric ether now almost exclusively used in this country.

The author's extensive statistical tables certainly tend to prove that the use of chloroform, both in surgery and midwifery, has been the means of saving many lives, and of averting incalculable suffering; but we still fear that the Doctor's enthusiasm and parental fondness for the anæsthetic in question, or, more properly speaking, for its benignant effects in almost all obstetrical cases, have made him take too favorable a view of its beneficial influences, and caused him entirely to overlook the fact that there is another side to the picture.

We sincerely hope that Dr. Simpson's most sanguine predictions of vast mercies in store for suffering humanity through the influence of anasthetics, may be more than realized, and that his name may descend to posterity and stand by the immortal Jenner's as that of a great benefactor of his species.

Still, we confess we have considerable doubt upon the subject; and we are sorry to admit that this doubt is strengthened, rather than otherwise, by Dr. S.'s article; it is too panegyrical; there is too much success; we have no per-contra; there is no dark side to the subject, and that is unnatural. It is true that the author is advocating a novelty, and one which has been violently opposed, and therefore, like all discoverers and improvers, he must be enthusiastic if he wishes to gain proselytes; and with many, no doubt, the style will have effect; but to thinking men, the sunshine must be mellowed by some shadow. No human agent can be perfect, or so nearly perfect as the author makes his favorite; therefore, these excessive praise make us pause and hesitate. Besides, though Dr. S. gets ver cleverly out of all difficulty, the list of accidents from the use of chloroform is somewhat startling, so much so that our best surgeons on this side of the water have totally abandoned its use, preferring ether, less potent perhaps, but far less dangerous; all this in the article before us is ignored, and this it is—great as is our admiration of Dr. Simpson—that would make us feel some hesitation in following his practice.

The answer to the religious objection to ameliorating the pains of parturition is admirably argued, and may be read with advantage by clergymen as well as physicians.

---

*Obstetrics: The Science and the Art.* By CHARLES D. MEIGS, M. D., Professor of Midwifery and the Diseases of Women and Children in Jefferson Medical College at Philadelphia, &c. *Third edition, revised. With one hundred and twenty illustrations.* Philadelphia: Blanchard & Lea. 1856.

It gives us much pleasure to welcome a new edition of this standard work. As a practitioner of vast experience, its respected author has enjoyed unusual opportunities of studying the art and science of obstetrics in all its lights and details; opportunities which he has appreciated and availed himself of with a zeal and industry evinced by very few anywhere, and which have deservedly placed him in the highest rank of authorities upon the subject. In his own country, he is confessedly at the head of his department, while abroad his works are received with such favor that no American is more frequently quoted, none certainly more complimented by the large space and free discussion which have been given to his peculiar views. Many of these views are stamped with great originality and power, and have won their way at once to general approval, while others are still considered by many, if not by a majority of the profession, as incorrect and untenable. As our readers, however, will find all these points fully discussed in some of our previous numbers, we shall not here dwell upon them.

Like all the other writings of Dr. Meigs, the present work derives much of its interest from the earnest manner and proper feeling in which it is written. In spite of its many faults of style, there is at once a power and ease in its narrative parts, a fervor and impressiveness, which place the author high in the ranks of descriptive writers. The just sense of duty, also, which the work everywhere inculcates, is calculated to exercise a most salutary influence upon the student's mind. He rises from its perusal with his nature elevated and refined, prepared morally as well as intellectually to meet the future duties and exigencies of his position. Such qualities, we need not say, are rarely found in medical works, the majority of which are so utterly barren of all grace of style and expression, so tied down by fancied restrictions, that they are as unattractive as the school-boy's lesson. To the traveller it is a gain to have even one stone removed from his weary path; how thankful should he be to find his whole journey macadamized.

---

*The Practical Anatomist: or The Student's Guide in the Dissecting Room.* By J. M. Allen, M. D., late Professor of Anatomy in the Medical Department of Pennsylvania College, &c. *With two hundred and sixty-six Illustrations.* Philadelphia: Blanchard & Lea. 1856.

The above admirable "Anatomist" was fully noticed (from advanced sheets kindly sent us by the author), in our October Number, 1855. We then observed "that the student who commences his early dissections with this assistant, will not have to regret the time and labor spent in the dissecting room." This opinion we take the present opportunity of repeating, as we believe it to be one of the most useful works upon the subject ever written. It is handsomely illustrated, well printed and will be found of convenient size for use in the dissecting room.

---

*An Introduction to Practical Chemistry, including Analysis.* By John E. Bowman, Professor of Chemistry in King's College, London, &c. *Second American from the second and revised London edition.* Philadelphia: Blanchard & Lea. 1856.

The above mentioned little work is intended as an introductory text-book to the science, and is well adapted to guide the student in his first attempt at experimental study.

It contains numerous directions for experiments to be made by the novice with the simple apparatus likely to be at his disposal, and choice is made of such experiments as will not only give him an insight into the nature of the substances, but also gradually lead him into the practice of actual analysis. The directions for qualitative and quantitative analysis in the latter part of the work are arranged after the system followed by Fresenius, and are well calculated to induce habits of neatness and accuracy, as well as to supply the needed information for the student's guidance. The tables of weights and measures, strength of solutions of acids and salts, solubility of substances, etc., are copious, and indeed far more extensive than those found in much larger works.

We commend it highly as a most pleasant and instructive companion for any one entering the paths of this science.

---

*Practical Anatomy. A new Arrangement of the London Dissector, with numerous Modifications and Additions, and with Illustrations.* By D. HAYES AGNEW, M. D., Lecturer on Anatomy, &c. Philadelphia: J. B. Lippincott & Co. 1856.

The preface informs us that the above work has been prepared with a single eye to the faithful economy of the student's time. The ability and experience of its author are vouchers that it is well suited to meet the design he had in view.

---

*Transactions of the American Medical Association, Instituted* 1847. Vol. IX. Philadelphia: Printed for the Association, by T. K. & P. G. Collins. 1856.

The above handsome volume of upwards of nine hundred pages was received two weeks ago. The various Reports, Histories of Epidemics, Prize Essay and other papers contained in it, are strong proofs of the great interest still taken in the "Association" by its members. From a cursory examination of its contents, we are satisfied that it is equal if not superior to any of its fellows.

---

*Sea Sickness: its Cause, Nature, Symptoms and Treatment, derived from experience and strict observation.* By M. NELKEN, Doctor Medecinæ of the Faculty of Medicine in Paris, France, &c. New York: Stringer & Townsend, 222 Broadway. 1856.

This little *brochure* comprises in its twenty-eight pages a clear though succinct account of the symtomatology, cause, nature and treatment of sea sickness. To these are added a few remarks upon regimen, a formulary of three pages, and a short account of the history and literature of the affection. Though somewhat pretentious in its style, it will repay the reader for the time passed in its perusal. The remedy found most generally useful by its author was the sulphate of morphia in half grain doses twice a day. In some instances this amount must be increased. The relief it affords does not usually last more than from twelve to twenty-four hours, and it is requisite to repeat the dose with the return of the symptoms.

If the treatment advised should afford anything like the relief it appears to have given in the writer's experience, it will prove a real god-send to those "that go down to the sea in ships."

